# Using Natural Gradients to Infer a Potential Response to Climate Change: An Example on the Reproductive Performance of *Dactylis Glomerata* L

**DOI:** 10.3390/biology1030857

**Published:** 2012-12-13

**Authors:** Matteo Dainese

**Affiliations:** Department of Land, Environment, Agriculture and Forestry, University of Padova, Viale dell’Università 16, 35020 Legnaro, Padova, Italy; E-Mail: matteo.dainese@unipd.it; Tel.: +39-049-827-2674; Fax: +39-049-827-2686

**Keywords:** elevational gradient, grassland, plant traits, precipitation, seed number, seed mass, temperature

## Abstract

An understanding of the climate conditions governing spatial variation in the reproductive performance of plants can provide important information about the factors characterizing plant community structure, especially in the context of climate change. This study focuses on the effect of climate on the sexual reproductive output of *Dactylis glomerata* L., a perennial grass species widely distributed throughout temperate regions. An indirect space-for-time substitution procedure was used. Sixty mountain populations of the same target species were surveyed along an elevation gradient, and then, a relevant climate model was used to infer a potential response to climate change over time. Within each population, information on the number of stems, seed number and seed mass were collected. Resource investment in reproduction (RIR) was quantified as seed number × seed mass. A clear variation was found in the reproductive performance of *D. glomerata* along the elevational gradient: RIR improved with increasing temperature. The best model included only one term: the maximum temperature of the warmest month. This study demonstrates that mountain ecosystems offer particularly good opportunities to study climate effects over relatively short distances and suggests that warming will enhance *D. glomerata*’s reproductive output throughout its elevational range. Furthermore, it can be hypothesized that a potential migration of *D. glomerata* toward higher altitudes may occur in response to accelerated climate change.

## 1. Introduction

Global warming has created a need for studies along climatic gradients to assess the effects of temperature on ecological processes [1]. Potential plant responses to global warming can be examined using observational investigations along environmental gradients [2,3]. As temperature decreases with elevation [4], these gradients serve as powerful study systems for answering questions on how ecological processes can be affected by changes in temperature and associated climatic variables [5,6]. Thus, mountain ecosystems offer particularly good opportunities to study climate effects over relatively short distances by using a space-for-time substitution [5]. Compared to latitudinal gradient studies, such an approach has the advantage of controlling the effects of factors that covary with latitude ,such as irradiation, photoperiod, land-use changes, historical and contemporary processes [1].

Plant responses to rising temperatures vary between species with different phenologies [3,7] and life forms [8]. For instance, Sherry *et al.* [7] found that tallgrass prairie plant species that flower before the summer heat peak tended to advance their phenology in response to warming, whereas later flowering species delayed their phenology. Further studies [8,9] have shown that graminoids, compared to forbs, generally exhibit higher plasticity to resource alterations, such as nitrogen enrichment and temperature increases.

Seed-related traits are considered important fitness parameters and have long been suggested as key traits influencing plant population dynamics and community structure [10,11,12,13]. The sexual reproductive phase in plants might be particularly vulnerable to the effects of global warming. The direct effect of temperature changes on the reproductive process has been previously documented, and recent data from other physiological processes that are affected by rising temperatures reinforce the concept that reproductive process are susceptible to a changing climate [14,15,16,17]. Unraveling the effect of temperature on possible future vegetation range shifts compared with other local abiotic and biotic environmental variables is important [17]. Moreover, climate change is of particular importance in predicting the outcome of a long-term relationship between plant life history (*i.e.*, reproductive traits) and climate change [3,17,18]. 

The use of long-term mean climatic variables in heterogeneous mountain systems (e.g., Worldclim [19]) has been shown to be of little relevance for processes that occur within a particular year or during a limited period of the year [1,4]. Therefore, such studies should focus on climatic information of the period under investigation, rather than considering long-term means. Furthermore, climate data extracted from local meteorological stations should be preferable to broad-scale climate databases [1].

To test the effect of altitudinal gradient and climate influence on reproductive traits, an herbaceous species was selected that tolerates a wide range of climatic conditions. *Dactylis glomerata* L. (Poaceae) is a perennial grass species widely distributed throughout temperate regions [20]. The species is widespread in hedgerows, meadows, pastures and rough grasslands [21]. In the Italian Alps, the species is distributed from lowlands to subalpine regions [22].

In this study, 60 populations of *D. glomerata* in the southeastern Alps, distributed along an elevational gradient (from 319 to 1,662 m a.s.l.), were evaluated. As it is unlikely that elevation *per se* underlies patterns in ecological traits and processes [23], population patterns are much more likely to be driven by correlated factors, such as energy, water availability and climatic seasonality. The main purpose of this study was, therefore, to test the shape and strength of relationships between the reproductive performance of *D. glomerata* and a range of climatic variables to better understand the potential impacts of climate change on its population dynamics and colonization capacities. To address this, a climate model was constructed using local meteorological stations, and climatic variables were extracted for the period under study [1]. Resource investment in reproduction (*i.e.*, the product of seed number and seed mass) was selected as a compound measure of reproductive performance. It represents an estimate of how many resources are used for sexual reproduction [3]. 

## 2. Results and Discussion

### 2.1. Results

#### 2.1.1. General Results

Climate variables were strongly correlated with each other due to their dependence on elevation (see Supplementary Figure S1). Environmental energy variables showed a negative relationship with elevation (Supplementary Figure S1a). Water availability variables showed two contrasting patterns along the elevational gradient: (i) a reduction of water availability during the driest season and (ii) increasing water availability during the wettest season (Supplementary Figure S1b). Climatic seasonality variables showed different patterns. Isothermality showed a positive relationship with elevation, while temperature seasonality and temperature annual range showed a negative relationship (Supplementary Figure S1c). Precipitation seasonality showed no significant relationship with elevation.

Resource investment in reproduction (RIR) per individual varied from a minimum of 44.9 mg to a maximum of 331.6 mg. RIR was higher in low-elevation populations (elevation ranged from 300 to 500 m a.s.l., and the averaged RIR was 230.2 ± 61.2 mg) than in higher elevation populations (elevation ranged from 1,400 to 1,600 m a.s.l., and the averaged RIR was 107.5 ± 20.8 mg).

#### 2.1.2. Relationships between Resource Investment in Reproduction and Climate Variables

**Figure 1:**
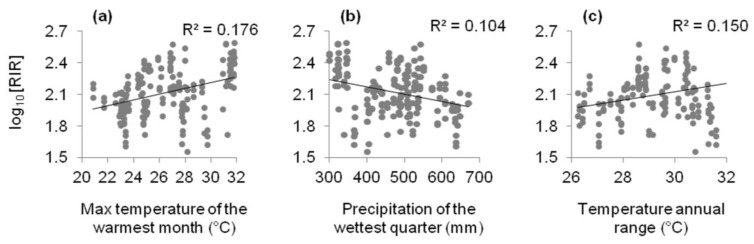
Relationships between log_10_ resource investment in reproduction (RIR) and selected climate variables: (**a**) maximum temperature of the warmest month (TEMP), (**b**) precipitation of the wettest quarter (PREC), and (**c**) temperature annual range (SEAS).

Among environmental energy variables, the maximum temperature of the warmest month (TEMP) was the best predictor of RIR (Table 1), although the other variables showed slight differences, except for minimum temperature of the coldest month and mean diurnal range. Environmental energy variables had a significant positive effect on the reproductive performance of *D. glomerata* along the elevational gradient. For instance, RIR consistently increased with TEMP (Figure 1a). The parameter estimate of the predictor TEMP in the equation of log-transformed RIR is 0.072 (Table 1), which means that RIR increased roughly with a factor 10^0.072^ = 1.18 mg for every 1 °C increase in TEMP. 

Precipitation of the wettest quarter (PREC) was the best single water-related predictor for the RIR, while the other water-related predictors explained lower variation in RIR (Table 1). Water availability had a significant negative effect on RIR. For instance, RIR consistently decreased with PREC (Figure 1b). The parameter estimate of the predictor PREC in the equation of log-transformed RIR is −0.003 (Table 1), which means that RIR decreased roughly with a factor 10^0.03^ = 1.07 mg for every 10 mm increase in PREC. 

Among climatic seasonality variables, the temperature annual range (SEAS) was the best predictor of RIR (Table 1) and showed a positive relationship with RIR (Figure 1c).

**Table 1:** Relationships between log_10_ resource investment in reproduction (RIR) and climate variables. All analyses were performed with linear mixed-effects models, including the random effect for district and site within district. The best predictor among each bioclimatic group (environmental energy, water availability and climatic seasonality) is shown in bold.

Variable	Intercept	β	*F*	*P*	*R^2^*
*Environmental energy*					
Max temperature of warmest month	2.971	0.072	12.413	<0.001	**0.176**
Mean temperature of wettest quarter	3.377	0.091	11.757	0.001	0.167
Mean temperature of warmest quarter	3.377	0.091	11.757	0.001	0.167
Annual mean temperature	3.817	0.111	12.190	<0.001	0.146
Mean temperature of driest quarter	4.396	0.109	11.518	0.001	0.146
Mean temperature of coldest quarter	4.396	0.109	11.518	0.001	0.146
Min temperature of coldest month	5.276	0.119	8.685	0.005	0.077
Mean diurnal range	3.819	0.096	1.273	0.264	0.020
*Water availability*					
Precipitation of wettest quarter	6.150	−0.003	9.412	0.003	**0.104**
Precipitation of wettest month	6.019	−0.007	6.379	0.014	0.102
Annual precipitation	6.252	−0.001	8.642	0.005	0.093
Precipitation of warmest quarter	5.154	−0.001	1.012	0.319	0.018
Precipitation of driest quarter	5.024	−0.001	0.277	0.601	0.004
Precipitation of coldest quarter	5.024	−0.001	0.277	0.601	0.004
Precipitation of driest month	4.776	−0.001	0.080	0.779	0.002
*Climatic seasonality*					
Temperature annual range	1.980	0.097	9.470	0.003	**0.150**
Temperature seasonality	2.036	0.004	8.278	0.006	0.137
Isothermality	6.519	−0.044	2.891	0.095	0.052
Precipitation seasonality	4.430	0.820	1.181	0.282	0.021

Pseudo-R^2^ (R^2^) calculated from components of the variance matrix is reported.

#### 2.1.3. Multi-Model Inference and Hierarchical Partitioning

Model selection indicated that no single best model was supported and that several candidate models were plausible in explaining patterns of RIR (Table 2). Therefore, as climatic variables were highly correlated, it was difficult to disentangle each effect. There was support for four models, including mostly TEMP. The proportion of explained variation by these models was relatively low (13%–16%). No significant interactions between climate variables were found. 

**Table 2:** Plausible candidate models (ΔAICc < 2) explaining resource investment in reproduction (RIR). The multi-model inference procedure included also the interactions between the predictors. As these interactions were never included in the set of plausible models, they are not shown in the table.

Variable importance	Coefficients	1^st^ mod.	2^nd^	3^rd^	4^th^
-	*R^2^*	0.18	0.15	0.14	0.13
-	ΔAICc	0	1.45	1.71	1.99
Σ *w_i_*	Model *w_i_*	0.52	0.25	0.22	0.14
-	Intercept	3.185	2.148	2.635	2.982
0.81	TEMP	0.060	-	0.045	0.070
0.23	PREC	-	-	-	−0.001
0.46	SEAS	-	0.092	0.035	

Models (columns) are ranked from left to right according to their ΔAICc. Variables (rows) are ranked according to their Σ*w_i_*. Parameter estimates, pseudo-*R^2^* (*R^2^*) calculated from components of the variance matrix and model weights (*w_i_*) are reported. Parameter estimates: TEMP, mean temperature of the coldest quarter; PREC, precipitation of the driest month; SEAS, temperature seasonality.

**Figure 2:**
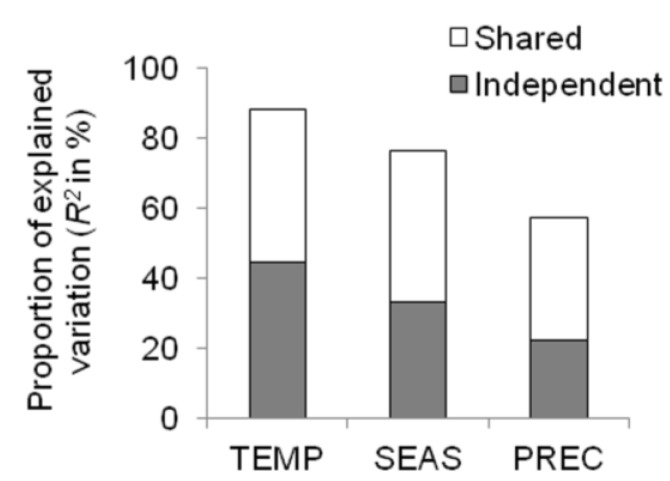
The independent contributions estimated from hierarchical partitioning of each bioclimate variable (TEMP, maximum temperature of the warmest month; PREC, precipitation of the wettest quarter; SEAS, temperature annual range) for the resource investment in reproduction (RIR). Variable ranking is conducted according to the size of the independent effect, *i.e.*, variable importance declines from left to right.

Results of the hierarchical partitioning reflected those ascertained by model selection (Figure 2). The variables ranking indicated that TEMP was the best predictor (44%), followed by SEAS (33%) and PREC (22%).

### 2.2. Discussion

An understanding of the climate conditions governing spatial variation in the reproductive performance of plants can provide important information about the factors characterizing plant community structure and influencing fitness in natural plant populations, especially in the context of climate warming [3,16,17,24]. As pointed out by Moles *et al.* [23], if we do not know how climate affects present‑day patterns in ecological traits and processes, then it will be very difficult indeed to predict the potential effects of climate change on the earth’s biota.

In the present study, a clear variation was found in the reproductive performance of *D. glomerata* populations along the elevational gradient. Resource investment in reproduction (*i.e.*, the product of mean seed number and mean seed mass per individual) decreased by 53% from warmer sites (where annual mean temperature is *ca.* 12.9 °C, and elevation ranged from 300 to 500 m a.s.l.) to colder sites (where annual mean temperature is *ca.* 6.6 °C, and elevation ranged from 1,400 to 1,600 m a.s.l.). Similarly, several authors [15,16,17,24] documented a lower reproductive performance in cold climate‑adapted populations (e.g., higher latitude/elevation) than in warm climate-adapted populations (e.g., lower latitude/elevation). As previously indicated by Dainese [17], climate change along the elevation gradient determines a morphological differentiation of reproductive traits in *D. glomerata*, with low-elevation individuals having larger inflorescences and larger seed masses than higher elevation individuals, meaning a higher reproductive output in warmer/longer growing seasons than in colder/shorter growing seasons. These results suggest that longer growing seasons and/or warmer climates in lower elevation sites promote higher photosynthetic rates, generating greater plant allocation toward reproductive performance than colder climates in higher elevations [17,24]. This means that cold climate-adapted populations are functioning on lower levels of photosynthesis compared to warm climate-adapted populations, determining a lower reproductive output [24].

Although the results of this study clearly indicate that reproductive performance of *D. glomerata* changes along the elevational/climatic gradient, recent studies have shown that this response is to some extent species-dependent. For instance, De Frenne *et al.* [3,15] reported a contrasting effect of temperature on reproductive performance in forest herbs due to their different phenology, distribution and life form. Hence, there is a need for studies that compare species responses to temperature, since results cannot just be transferred from model species [3].

Considering the influence of climate variables on the reproductive performance of *D. glomerata*, this study shows a greater importance of environmental energy than water availability, confirming the primary importance of temperature in mountain systems [4]. The negative relationship between water availability during the growing season and reproductive performance might be due to the co-variation between elevation/temperature and precipitation.

Bioclimatic variables that provide information about climatic conditions during the harshest times of the year for plant performance (e.g., minimum temperature of the coldest quarter or precipitation of the driest month), are relatively weakly correlated with resource investment in reproduction. Moles *et al.* [23] found a primary influence of variables that capture information about the quality of the growing season compared to variables that capture information about difficult times, when growth is low or entirely ceased. Therefore, it would be advisable to take into account a range of climatic variables, especially those that consider the growing season, when studying potential plant responses to climate change.

However, in interpreting elevational gradient studies, one must consider that besides climate change, the variation of elevation can also modify local conditions (*i.e.*, plant competitor, seed predator density, herbivorous species) [4,5]. The cascading effects of change in local conditions could indirectly modify the reproductive performance of *D. glomerata.* Thus, the observed variation of the resource investment in reproduction of *D. glomerata* may be attributable to both direct and indirect effects. Moreover, in a context of climate change, an increment in reproductive performance of competitive species, such as *D. glomerata*, could affect plant population dynamics and the community structure of neighboring plant communities, according to the total competition hypothesis [17,25,26]. As hypothesized by Dainese [17], the higher reproductive performance of competitive species may allow them to outcompete smaller and less competitive species.

## 3. Experimental Section

### 3.1. Case Study and Data Collection

To generate a climatic gradient, an elevation gradient was used as a ‘space for time substitution’. The dataset from Dainese [17] was used to test the relationship between the reproductive performance of *D. glomerata* and a range of climatic variables. In a previous study, Dainese [17] has used long-term mean climatic variables and considered only limited annual trends (*i.e.*, annual mean temperature and annual precipitation) to test the influence of climate change on the reproductive performance of *D. glomerata*. In this study, a climate model was constructed using local meteorological stations and considering monthly climate data for the study year and the two previous years (2006–2008). Moreover, several bioclimatic variables were generated to take into account the quality of the growing season, as well as annual trends and limiting environmental factors. In Dainese [17], 60 grasslands were sampled along an elevational gradient (from 319 to 1,662 m a.s.l.) on the southern border of the European Alps (Province of Trento—NE Italy; N45°55'–46°28'; E11°10'–11°53') (see Supplementary Figure S2). The grasslands were distributed across three different districts: (i) Valsugana, (ii) Primiero, and (iii) Fiemme and Fassa Valley. The mean annual temperature at the minimum altitude considered (319 m a.s.l.) was 12.9, and 5.4 °C at the maximum altitude (1,662 m a.s.l.). The mean annual rainfall varied from 700 to 1,500 mm year^−1^. 

At each site in 2008, three 1 m × 1 m plots were randomly located, and all fertile stems of *D. glomerata* were collected and counted. Seeds were extracted and weighed for each plot (*n* = 180). The total seed mass per individual was determined by dividing the seed production per plot by the total number of fertile stems per plot. One hundred seeds from each plot were then randomly sampled and weighed and the mean seed mass calculated. Finally, the total number of seeds per individual was calculated as the total seed mass per individual divided by the mean seed mass. 

### 3.2. Climate Model

Temperature and precipitation data were extracted from 31 local meteorological stations (with a minimum mean distance between meteorological stations and sampling sites of 4.9 km), covering an elevation range between 182 and 2,063 m a.s.l. (see Supplementary Figure S2). Averaged monthly precipitation and temperature for the period 2006–2008 were used to generate 19 bioclimatic variables, which represent annual trends, seasonality and extreme or limiting environmental factors. All bioclimatic variables were grouped into three categories: (i) environmental energy, including annual mean temperature, mean diurnal range, maximum temperature of the warmest month, minimum temperature of the coldest month, mean temperature of the wettest quarter, mean temperature of the driest quarter, mean temperature of the warmest quarter and mean temperature of the coldest quarter; (ii) water availability, including annual precipitation, precipitation of the wettest month, precipitation of the driest month, precipitation of the wettest quarter, precipitation of the driest quarter, precipitation of the warmest quarter and precipitation of the coldest quarter; and (iii) climatic seasonality, including temperature annual range, isothermality, temperature seasonality and precipitation seasonality. 

Since the environmental energy variables were highly correlated with elevation (average *r* = −0.92; range: −0.97 ÷ −0.70), except for mean diurnal range, ordinary kriging with external drift was used as the interpolation method [27]. First, the fitted environmental energy variables were estimated from a simple regression with elevation. Then, the residuals were interpolated using ordinary kriging and, subsequently, summed with the fitted temperature from the regression with elevation. The same procedure was also used for some water availability variables due to their strong correlation with elevation, such as annual precipitation (*r* = 0.65), precipitation of the wettest month (*r* = 0.63) and precipitation of the wettest quarter (*r* = 0.78). Instead, the other variables, which were not correlated with elevation, were interpolated using ordinary kriging. The geostatistical interpolations were computed using the Geostatistical Analyst extension for ArcGIS 9.3 (ESRI).

### 3.3. Data Analysis

Seed number and seed mass were used to calculate a compound measure of reproductive success: ‘resource investment in reproduction’ (RIR), as the product of mean seed number and mean seed mass per individual [3,17].

Due to the hierarchical nature of the data, linear mixed models (LMMs) were constructed for all regression analyses to account for dependencies within hierarchical groups through the introduction of random effects, therefore maximizing the statistical power. Models included bioclimatic variables as fixed effects and district and site-within-district as random effects. The models were validated by the analysis of residuals, to assess homogeneity and to verify normality [28]. Response variables were log-transformed to satisfy assumptions of normality. Furthermore, to avoid problems of heterogeneity, a variance model with different variances for each level of a stratification variable (in this case district) was applied when necessary [28]. Models were estimated using the ‘nlme’ package [29] in R, version 2.14.1 [30] with the restricted maximum-likelihood (REML) estimation method. The strength of the association between bioclimatic variables and RIR was calculated from the variance components of the mixed-effects models using a level-1 pseudo-*R^2^* statistics [31].

A combined model was developed using the following methods: (i) the best individual predictor for RIR of a bioclimatic group was selected in the model; (ii) to avoid the multi-co-linearity between the predictors of the same environmental category, only one variable from each category was used in the model; and (iii) all the possible combinations of predictors following the above criteria were examined using a multi-model inference within an information theoretic framework [32]. The information-theoretic approach compared the fit of all possible candidate models obtained by the combination of the predictors using second-order Akaike’s information criterion (AICc). The AICc is a measure of relative model fit, proportional to the likelihood of the model and the number of parameters used to generate it. The best fitting model is the one with the lowest AICc. In a set of n models, each model *i* can be ranked using its difference in AICc score with the best-fitting model (ΔAICci = AICc_i_–AICc minimum). The difference in AICc values indicates the relative support for the different models. A model is usually considered plausible if its ΔAICc is below 2 [32]. For each model *i*, an Akaike’s weight (*w_i_*) was calculated, which is the probability that model *i* would be selected as the best fitting model if the data were collected again under identical circumstances [32]. Akaike’s weight should be interpreted as a measure of model selection uncertainty. The multi-model inference analyses were performed using the ‘MuMIn’ package [33] implemented in R.

Finally, hierarchical partitioning (HP) [34] was used to evaluate the relative importance of predictors, given that the use of Akaike’s model weights has been recently criticized in cases of co‑linearity between the variables included in model selection [35]. HP addresses the presence of co‑linearity by determining the independent contribution of each explanatory variable to the response variable and separates it from the joint contribution, resulting from the correlation with other variables [34]. HP was conducted using the ‘hier.part’ package [36] for R. A normal error distribution and *R^2^* as a measure of goodness-of-fit were used.

## 4. Conclusions

This study demonstrates that mountain ecosystems offer particularly good opportunities to study climate effects over relatively short distances by using a space-for-time substitution [5]. Some limitations of elevation gradient studies are noteworthy. Because there is no ‘standard mountain’, any data collected along elevational gradients reflects the combined effect of local conditions and general altitude phenomena [6]. Hence, the response of plant performance could be different in other mountain systems.

Although previous studies have reported contrasting responses of temperature and climate warming on reproductive output [3,18], the results of this study highlight an increasing trend in the reproductive performance of *D. glomerata* along the climatic gradient, suggesting that warming will enhance *D. glomerata*’s reproductive output. This higher reproductive performance could improve the establishment success of *D. glomerata*, since an increase in mass per seed has been shown to correlate with higher seed germination, growth rate and survival [37,38,39,40]. Furthermore, it can be hypothesized that the potential migration of *D. glomerata* toward higher altitudes could occur in response to accelerated climate change. This analysis should be extended to a larger set of grassland plant species to understand how different functional species groups react to temperature changes and to find common responses among species. 

## Supplementary Files

Supplementary File 1 Supplementary Information (PDF, 250 KB)
