# Supplementary material for: Using Natural Gradients to Infer a Potential Response to Climate Change: An Example on the Reproductive Performance of Dactylis Glomerata L
Source: Biology (Basel). 2012 Dec 13;1(3):857–68. doi: 10.3390/biology1030857 (PMC4009817; doi:10.3390/biology1030857)

Figure S1. Scatterplots of elevation *versus*: (**a**) environmental energy variables, (**b**) water availability and (**c**) climatic seasonality. Solid and dashed lines show the results of linear regression. Solid lines represent slopes that are significantly different from zero at alpha = 0.05.

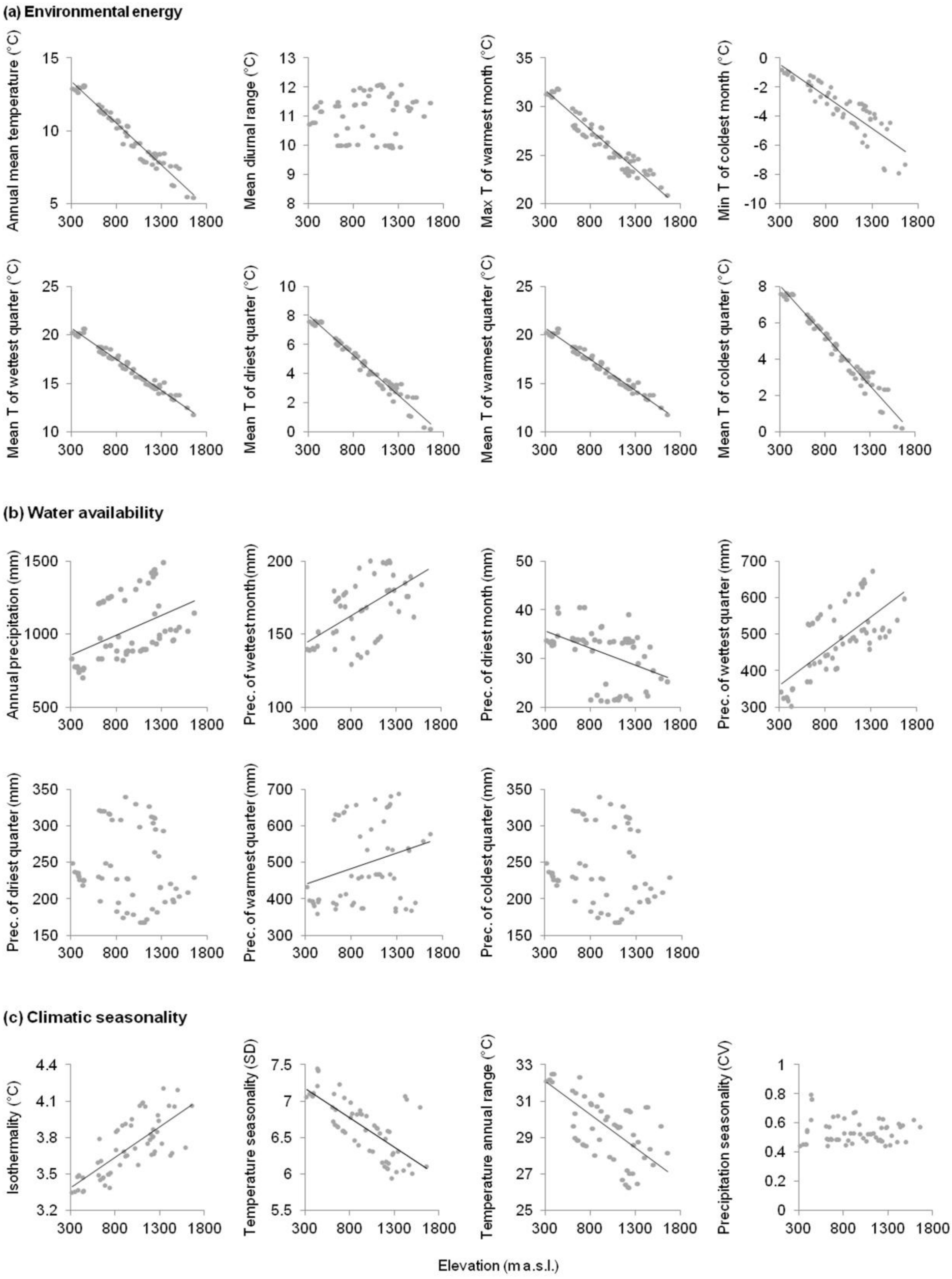

Figure S2. Distribution of (●) the *Dactylis glomerata* populations (n = 60) sampled and (▲) the meteorological stations (n = 31) in the eastern province of Trento (NE Italy).

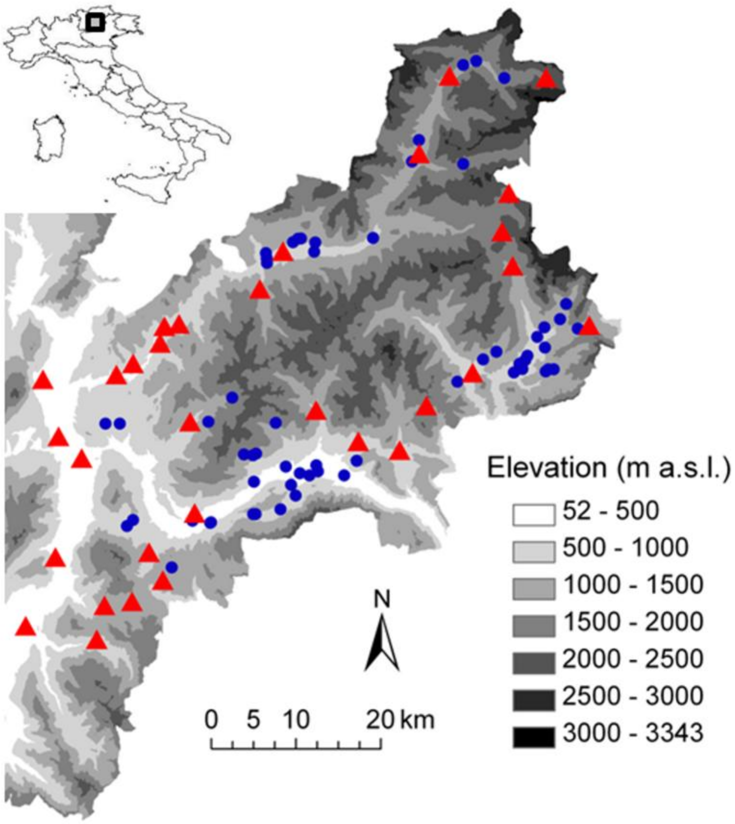

Supplement: Supplementary File 1 — Supplementary Information (PDF, 250 KB) [file biology-01-00857-s001.pdf]
